# Glycogen synthase kinase-3β inhibitor promotes the migration and osteogenic differentiation of rat dental pulp stem cells via the β-catenin/PI3K/Akt signaling pathway

**DOI:** 10.1016/j.jds.2021.09.035

**Published:** 2021-10-16

**Authors:** Huilan Xie, Yi Lin, Fang Fang

**Affiliations:** Department of Stomatology, Fujian Provincial Hospital, Fuzhou, Fujian, China

**Keywords:** Glycogen synthase kinase-3β inhibitor, Dental pulp stem cells, Migration, Osteogenic differentiation, PI3K

## Abstract

**Background/purpose:**

Glycogen synthase kinase-3β (GSK3β) inhibitor enhances bone formation, while dental pulp stem cells (DPSC) are potentially used to repair bone defects. The present study aimed to investigate the effect of AR-A014418 (AR, a specific glycogen synthase kinase-3β inhibitor) on the migration and osteogenic differentiation of rat-derived dental pulp stem cells (rDPSCs), and further explore the underlying mechanism.

**Materials and methods:**

rDPSCs were isolated from rats, and then cultured with different concentrations of AR with or without LY294002 (a PI3K inhibitor). Then, cell viability, migration, osteogenic differentiation, and the involvement of PI3K pathway were detected by CCK-8 assay, Transwell assay, Alizarin Red S Staining, Alkaline phosphatase (ALP) assay, Western blot, and RT-PCR, respectively.

**Results:**

Our present study demonstrated that AR of various concentrations (1 μM, 2.5 μM, and 5 μM) not only promoted the rDPSC proliferation and migration, but also increased calcium deposition, the activity of alkaline phosphatase (ALP), and levels of osteogenic markers (RUNX2, OPN, OCN, and OSX) in rDPSCs. It was also found that the administration of AR resulted in an increase in the expression level of p-GSK3β (Ser), β-catenin, p-PI3K, and p-Akt, and a reduction in p-GSK3β (Tyr216). Furthermore, PI3K inhibitor LY294002 abrogated the enhanced cell migration and osteogenic differentiation of rDPSCs induced by AR.

**Conclusion:**

Our results provide evidence that AR significantly promotes migration and osteogenic differentiation of rDPSCs by activating β-catenin/PI3K/Akt signaling pathway.

## Introduction

As all known, stem cells can self-renew and differentiate into other cell types. In the last decade, mesenchymal stem cells (MSCs) have shown great potential in the field of regenerative medicine due to their capacities of self-renewal and multilineage differentiation.^1^^,^^2^ MSCs could be isolated from numerous tissues including adipose tissues,^3^ bone marrow,^4^ skeletal muscle,^5^ and dental tissues.^6^ In 2004, Seo et al.^7^ initially isolated and identified dental pulp stem cells (DPSCs) from the human pulp tissues. Since DPSCs not only display a MSC-like property but also can be conveniently harvested, they are considered promising stem cell sources for clinical use.^8^ Extensive basic studies proved the potent potential of DPSCs in the regeneration of bone.^9^^,^^10^ Additionally, a previous clinical study confirmed the safety and therapeutic effects of DPSCs in treating severe periodontal defects.^11^ It is undoubted that the osteogenic differentiation capacity of DPSCs is crucial for bone regeneration. Besides, the migration of endogenous or exogenous DPSCs to bone injury or loss sites is also significant for bone regeneration.^12^ Therefore, enhancement of osteogenic differentiation and migration of DPSCs is an important avenue in improving the therapeutic efficacy of DPSC-based therapy.

In recent years, increasing studies have found that some small molecules show osteogenic function via diverse signaling mechanisms.^13^, ^14^, ^15^ Given that it is easy to synthesize small molecules, which are non-immunogenic and cost-effective, it seems an attractive choice for directing osteogenic differentiation and migration of PDLSCs. Glycogen synthase kinase-3 (GSK3) is a serine–threonine protein kinase containing two isoforms (GSK3α and GSK3β), which is an important component of various key signaling pathways, including insulin, mTOR, NF-κB, and Wnt/β-catenin.^16^ Many studies have implicated the Wnt signaling pathway in the lineage differentiation and migration of MSCs.^17^^,^^18^ It is well known that GSK3β inhibitors can activate the Wnt signaling pathway by decreasing the degradation of β-catenin. Hence, GSK3β inhibitors may provide a new therapeutic strategy for DPSC-based bone regeneration. In the past decades, a number of small molecule GSK3β inhibitors have been identified;^19^, ^20^, ^21^ however, more than half of the published inhibitors not only target GSK3β, but also affect cyclin-dependent kinase 2 (cdk2) or cdk5.^22^ In 2003, Bhat et al.^23^ identified an amino thiazole in a high-throughput biochemical screening using purified recombinant GSK3β, which was named as AR-A014418 (AR). AR can inhibit GSK3β activity in an ATP-competitive way, with high specificity for GSK3β as it does not affect the activity of other 26 protein kinases, especially cdk2 and cdk5 (IC50 > 100 μM) that are inhibited by the majority of GSK3 inhibitors.^23^ Several studies reported that AR had potent activities against diverse types of cancer with no adverse effects in rodents.^24^^,^^25^ However, to our knowledge, the role of AR in migration and osteogenic differentiation of DPSCs has been rarely studied before.

The aim of this study is to investigate the effects of GSK3β inhibitors, AR, on migration and differentiation of rat DPSCs (rDPSCs) into osteoblasts *in vitro*, and preliminarily explore the underlying molecular mechanisms.

## Materials and methods

### rDPSCs isolation and characterization

Sprague–Dawley rats (3 months, SPF grade) were purchased from Guangdong Medical Laboratory Center (Guangzhou, China), and rDPSCs were isolated from the dental pulp of incisors of rats, as Alksne et al.^26^ previously described. The isolated rDPSCs were cultured in DMEM supplemented with 10% FBS and 1% penicillin/streptomycin at 37 °C in a 5% CO_2_ incubator. The third rDPSC passage was subjected to characterization, which included identification of the cell surface markers and multidirectional differentiation of rDPSCs. Every procedure was approved by the Animal Care and Use Committee of Fujian Provincial Hospital.

### Osteogenic and adipogenic differentiation

For osteogenic differentiation, 5 × 10^4^ rDPSCs were maintained in cultured media containing 10 mM β-glycerol phosphate, 50 μg/mL l-ascorbic acid, 10 nM dexamethasone, and 2 mM glutamine for 7 or 14 days. During the cultivation, the media were renewed three times every week.

For adipogenic differentiation, 5 × 10^4^ rDPSCs were maintained in cultured media containing 5 μg/mL insulin, 0.5 mM 3-isobutyl-1-methylxanthine, and 1 μM dexamethasone for 14 days. During the cultivation, the media were renewed every other day.

### Alizarin red S (ARS) staining

ARS staining was conducted to confirm osteogenic differentiation and assess calcium-rich deposits in rDPSCs. In brief, after incubated with osteogenic media for 14 days, rDPSCs were fixed with 4% paraformaldehyde (PFA) for 15 min and subsequently washed with PBS. 2% ARS solution was added and incubated for 15 min. Thereafter, the stained rDPSCs were observed under an optical microscope. For quantitative evaluation, ARS was dissolved in 5% perchloric acid by measuring absorbance at 562 nm under a microplate spectrophotometer.

### Oil red O staining

As a fat-soluble diazo dye that can stain lipid droplets in cells, Oil Red O is usually utilized to confirm the adipogenic differentiation of MSCs. Briefly, rDPSCs were fixed with 4% PFA, followed by staining with Oil Red O solution in isopropanol for 15 min. Finally, differentiated oil red O-positive rDPSCs were observed and imaged under a microscope.

### Western blot

rDPSCs were lysed in RIPA buffer (Abcam, Cambridge, MA, USA) on ice to extract total protein. The total protein was separated by SDS-PAGE gels and electrophoretically transferred onto PVDF membranes, which were subsequently blocked in skimmed milk (12%) for 2 h. The membranes were incubated overnight at 4 °C with primary antibodies, and subsequently rinsed three times before incubation with the secondary antibody. Finally, protein bands were visualized using ECL Substrate Kit (Abcam), and quantified using Image J software (Bethesda, MD, USA). The information of antibodies used in this study was described in Supplementary Table S1.

### CCK-8 assay

rDPSCs were cultured in media containing different concentrations (0, 1, 2.5, 5, 10, and 20 μM) of AR (Sigma–Aldrich; A3230) for 1 day, 3 days, 5 days, and 7 days, respectively. At the indicated time points, a CCK-8 reagent (10%, v/v) was added to each well. Finally, the absorbance at 450 nm was measured under a microplate reader (Bio-Rad, Hercules, CA, USA) after additional 2-h incubation.

### Transwell assay

Briefly, 700 μL cultured media were added to the bottom chamber. Then, 200 μL rDPSCs suspension (serum-free) with different treatments was added to the top chamber with a filter membrane (8 mm pores), followed by 24-h incubation. The cells which migrated to the bottom chamber were fixed and stained. They were then photographed under a microscope.

### Alkaline phosphatase (ALP) assay

After incubated with osteogenic media for 7 days, the ALP activity of rDPSCs was determined using colorimetric Alkaline Phosphatase Assay Kit (Abcam). In short, rDPSCs were incubated with ALP substrate for 15 min in the dark. Then, the reaction was stopped by 2 M NaOH. The absorbance of the product was detected at 405 nm under a microplate reader.

### RT-PCR

Total RNA was extracted from rDPSCs by using TRIzol reagent (Life Technologies, Carlsbad, CA, USA). Thereafter, RT-PCR analysis was conducted as described previously^27^ with the following primer sequences: RUNX2 (F: 5-‘GATAACCTGGATGCCGTCGTG-3′, R: 5′-CAGCCTAGCCAGTCGGATTTG -3′), OPN (F: 5-‘CAGGTTCACCCCATTCTCC-3′, R: 5′-AAATTATCCGGGCGTGGT-3′), OCN (F: 5-‘CAGCGTTATGAGATCAAGATGACCA-3′, R: 5′-AGTGATGTGCAAGAGTCCATCCTG-3′), OSX (F: 5-‘GCTTTTCTGTGGCAAGAGGTTC-3′, R: 5′-CTGATGTTTGCTCAAGTGGTCG-3′ GADPH (F: 5-‘GAGAAGTATGACAACAGCCTC-3′, R: 5′-ATGGACTGTGGTCATGAGTC-3′). The subsequent quantification was carried out using the QuantiTect SYBR Green PCR Kit (Toyobo, Osaka, Japan) on a 7500 Real-Time PCR System (Applied Biosystems, Förster, CA, USA). GAPDH was selected as an internal control for normalization in this study.

### Statistical analysis

All data were expressed as mean ± standard deviation (SD) of three independent experiments. The Student's t-test was used for comparison between two groups. For multiple-group condition, one-way ANOVA was performed with Bonferroni's method. P < 0.05 indicated significant difference.

## Results

### rDPSC characterization

**Figure 1:**
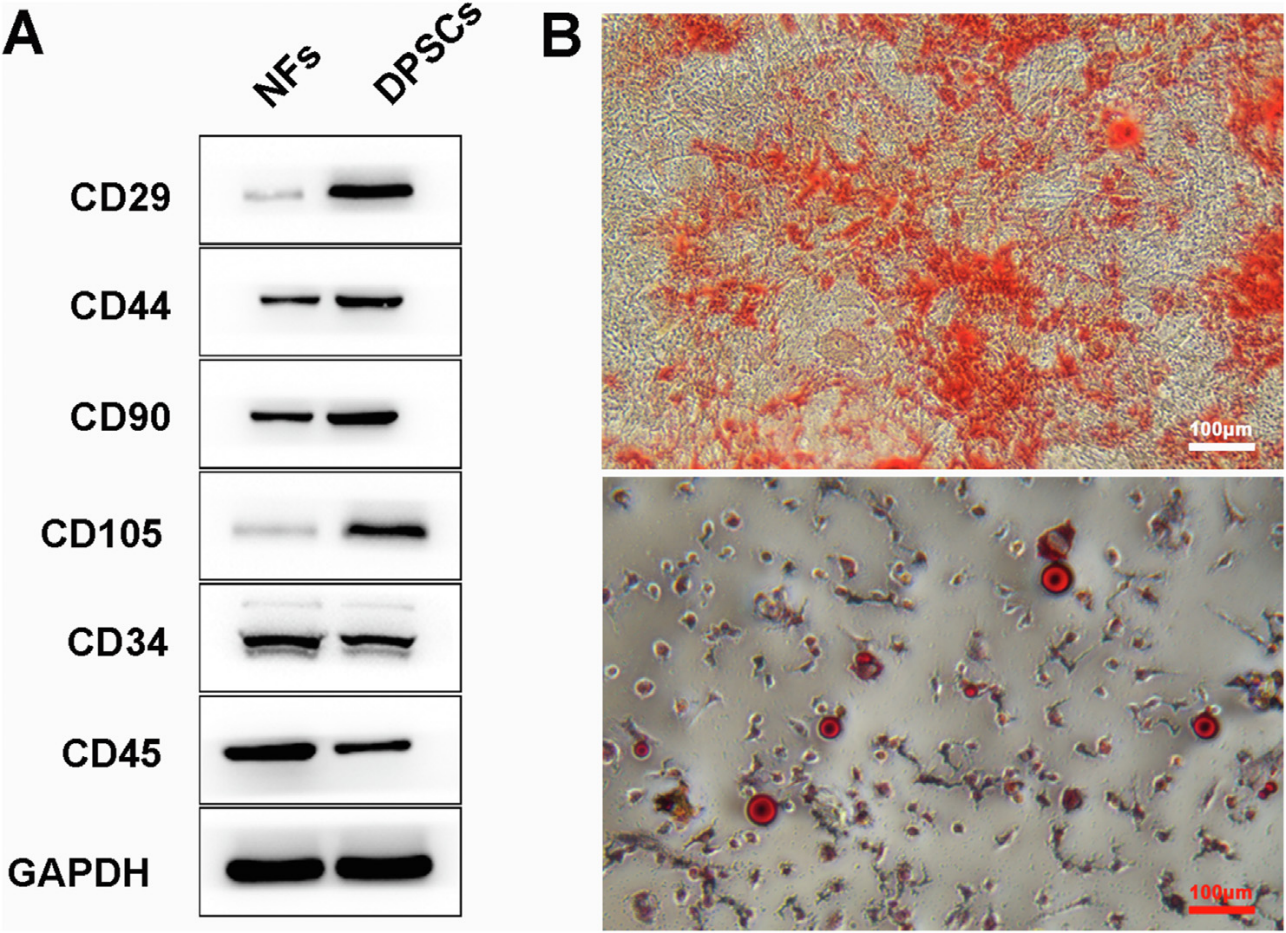
**Characterization of rDPSCs. (A)** Identification of stem cell markers in rDPSCs using Western blot. Normal fibroblasts (NFs) without stemness were used as control. **(B)** Osteogenic differentiation (top) and adipogenic differentiation (bottom) of rDPSCs detected by ARS and Oil Red O staining, respectively.

rDPSCs were isolated from incisors of rats, of which surface antigens were characterized using Western blot. Compared with non-DPSCs, CD29, CD44, CD90, and CD105 were obviously up-regulated, while CD34 and CD45 were down-regulated in the isolated rDPSCs (Fig. 1A). ARS staining and Oil red O staining respectively confirmed the role of rDPSCs in osteogenic differentiation and adipogenic differentiation (Fig. 1B). All these data suggested successful isolation of rDPSCs.

### AR enhances migration and osteogenic differentiation of rDPSCs

**Figure 2:**
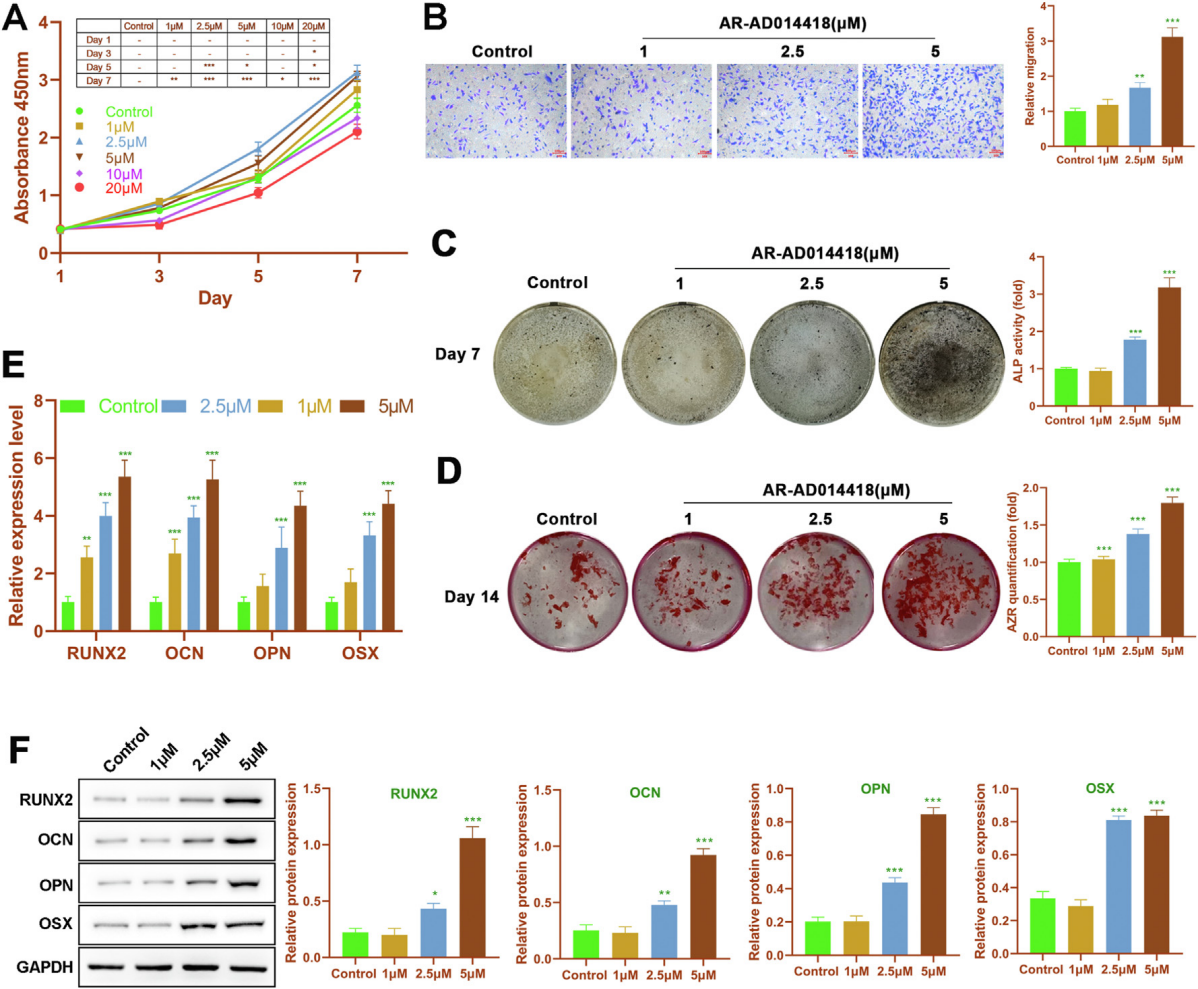
**The effect of AR-A014418 on cell proliferation, migration, and osteogenic differentiation of rDPSCs. (A)** The cell viability of rDPSCs determined by CCK-8 assay after 1-, 3-, 5-, and 7-day treatment. **(B)** The migration of rDPSCs detected by Transwell assay. **(C)** The ALP activity of rDPSCs. **(D)** The calcium deposits in rDPSCs visualized by using ARS staining. The expressions of osteogenic markers (RUNX2, OPN, OCN and OSX) were detected by **(E)** RT-PCR and **(F)** Western blot, respectively. ∗∗P < 0.01 and ∗∗∗P < 0.005, versus control.

Using the CCK-8 kit, the effect of AR (0, 1, 2.5, 5, 10, and 20 μM) on rDPSCs proliferation was observed (Fig. 2A). On day 1 and day 3, there was no significant discrepancy in rDPSCs proliferation between groups. After 5-day and 7-day incubation, both 10 and 20 μM of AR suppressed proliferation of rDPSCs. Notably, after 5-day and 7-day incubation, 15 μM, 2.55 μM and 5 μM of AR enhanced proliferation of rDPSCs. Hence, 15 μM, 2.55 μM and 5 μM of AR were selected to investigate the effect of AR on rDPSCs migration and osteogenic differentiation. As shown in Fig. 2B, the migratory capacity of rDPSCs was promoted by AR in a concentration-dependent way.

The biological role of AR in osteogenic differentiation of rDPSCs was also explored using ALP assay, and ARS staining, and the expression of osteogenic markers (RUNX2, OPN, OCN, and OSX) was determined. The results showed that the ALP activity of rDPSCs in 2.5 μM and 5 μM groups was significantly higher than that in the control group after osteogenic induction for 7 days (Fig. 2C). Similar to the result of the ALP assay, the ARS staining showed there was no significant difference between the control and 1 μM groups (Fig. 2D). Besides, the deposition of calcium induced by osteogenic media in rDPSCs was significantly increased by 2.5 μM and 5 μM of AR (Fig. 2D). Moreover, both 2.5 and 5 μM of AR significantly elevated the expression of RUNX2, OPN, OCN, and OSX in rDPSCs at mRNA and protein levels (Fig. 2E and F). Taken together, our results indicated that AR plays a positive role in migration and osteogenic differentiation of rDPSCs.

### AR promoted migration and osteogenic differentiation of rDPSCs via the Wnt/β-catenin/PI3K/Akt signaling

**Figure 3:**
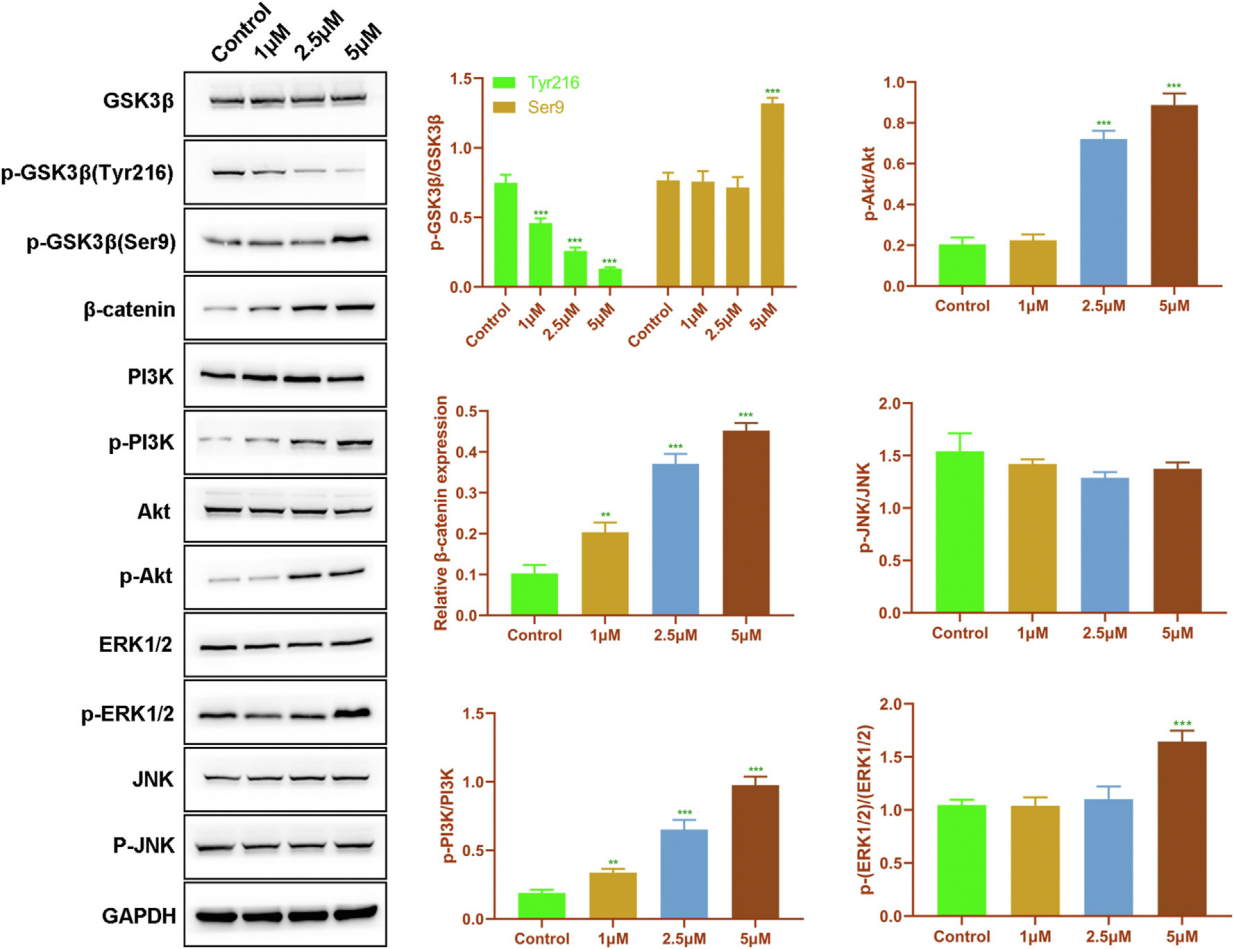
**The effect of AR-A014418 on the Wnt/β-catenin, PI3K/Akt, ERK1/2 and JNK signaling pathways in rDPSCs**. ∗∗P < 0.01 and ∗∗∗P < 0.005, versus control.

To preliminarily explore the potential mechanisms of AR acting in migration and osteogenic differentiation, the effect of AR on the activity of Wnt/β-catenin, PI3K/Akt, ERK1/2, and JNK signaling pathways in rDPSCs was investigated. The results were depicted in Fig. 3. Compared with the control group, AR treatment decreased the phosphorylation of GSK3β at Tyr216 along with an increase in the expression of β-catenin. Besides, the phosphorylation of GSK3β at Ser9 was increased by the treatment of 5 μM AR. These data suggested that AR could reduce the activation of GSK3β, thereby increasing the expression of β-catenin to activate Wnt/β-catenin signaling. In addition, the treatment of AR also increased the phosphorylation of both PI3K and Akt in a concentration-dependent way. The phosphorylation of ERK1/2 was obviously increased when the concentration of AR reached 5 μM. However, there was no obvious difference in the phosphorylation of JNK among all groups. Collectively, our results suggested that the Wnt/β-catenin/PI3K/Akt signaling was involved in the effect of AR on migration and osteogenic differentiation of rDPSCs.

### Inhibiting PI3K reversed the effect of AR on migration and osteogenic differentiation of rDPSCs

**Figure 4:**
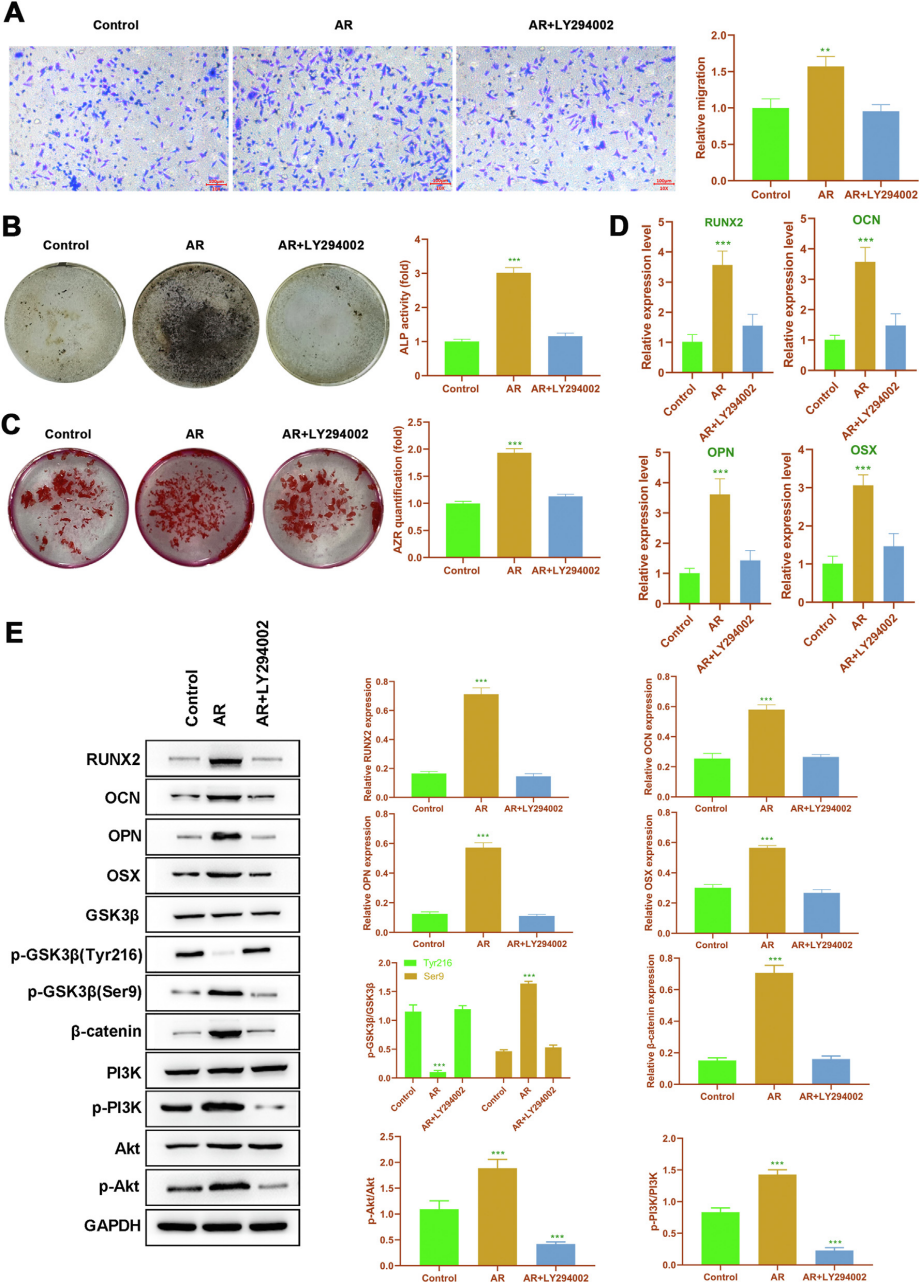
**PI3K inhibition by LY294002 blocked the promoting role of AR-A014418 in migration and osteogenic differentiation of rDPSCs. (A)** The migration of rDPSCs detected by Transwell assay. **(B)** The ALP activity of rDPSCs. **(C)** The calcium deposits in rDPSCs visualized by using ARS staining. **(D)** The mRNA expressions of RUNX2, OPN, OCN and OSX were detected by RT-PCR. **(E)** The expressions of osteogenic markers and Wnt/β-catenin/PI3K/Akt signaling determined by Western blot. ∗∗P < 0.01 and ∗∗∗P < 0.005, versus control.

To confirm the role of PI3K/Akt signaling in the function of AR in rDPSCs, rDPSCs were pretreated with LY294002 (15 μM), a specific PI3K inhibitor, for 1 h before AR treatment (5 μM). LY294002 significantly weakened the migration ability of AR-treated rDPSCs (Fig. 4A). ALP assay and ARS staining indicated that LY294002 effectively reversed the increased osteogenic differentiation induced by AR (Fig. 4B and C). Additionally, LY294002 also significantly decreased the expressions of osteogenic markers in AR-treated rDPSCs at both mRNA and protein levels (Fig. 4D and E). We further investigated the effect of LY294002 interference on Wnt/β-catenin/PI3K/Akt signaling in AR-treated rDPSCs. As expected, the treatment of LY294002 noticeably decreased expression of p-PI3K and p-Akt in AR-treated rDPSCs. In the meantime, LY294002 also effectively suppressed the decrease of p-GSK3β (Tyr216) and increase of p-GSK3β (Ser9) and β-catenin induced by AR treatment. In short, these data suggested that PI3K/Akt signaling pathway was involved in the role of AR in migration and osteogenic differentiation of rDPSCs.

## Discussion

DPSCs are an attractive source of MSCs for regenerative medicine due to their relative accessibility and lack of ethical controversy.^28^^,^^29^ It has been demonstrated that DPSCs display greater proliferative potential than bone MSCs.^30^ During the application of DPSCs for bone regeneration, it is required for DPSCs to expand and migrate to the bone damaged/defect area and differentiate into osteoblasts. Hence, it is important to explore a strategy for promoting DPSCs migration and osteogenic differentiation in DPSC-based therapy. In this study, it was demonstrated that AR, an inhibitor specific to GSK3β, may promote cell migration and osteogenic differentiation via specifically activating Wnt/β-catenin/PI3K/Akt signaling pathways in rDPSCs.

In an attempt to determine the ideal concentration range of AR on the cell proliferation of rDPSCs, a CCK-8 assay was carried out on rDPSCs after treated with diverse concentrations of AR. In our study, after 7 days of incubation, AR suppressed cell viability of rDPSCs at a concentration of 10 up to 20 μM, while no more than 5 μM promoted the cell viability. Based on the results, a concentration range of 1–5 μM was chosen for the subsequent experiments. Thereafter, our data demonstrated that the migratory ability of hDPSCs was enhanced by 2.5 and 5 μM of AR. Previous studies indicated that GSK3 inhibitors potentially promoted osteogenic differentiation of several MSCs.^31^, ^32^, ^33^ Similar to the literature, our study showed that the indicated concentrations of AR enhanced ALP activity and calcium deposition. Moreover, both the mRNA and protein expressions of RUNX2, OPN, OCN, and OSX were up-regulated in a concentration-dependent way. RUNX2 is a crucial transcription factor involved in osteoblast differentiation, which regulates expression of many osteogenesis-related genes.^34^ Takahashi^35^ indicated that the overexpression of RUNX2 induced differentiation of preadipocytes into functional osteoblasts. RUNX2 enhances the expression of OSX,^36^ thereby inducing the expression of OCN and OPN.^37^ These data collectively revealed that AR promoted migration and osteogenic differentiation of rDPSCs.

It is well known that Wnt/β-catenin signaling is involved in tissue generation, regeneration, as well as self-renewal.^38^ Given that GSK3β is one of the key components of Wnt/β-catenin signaling, we assessed the effect of AR on the activity of the Wnt/β-catenin pathway. The activity of GSK3β is down-regulated by phosphorylation at the Ser9 site, while up-regulated by phosphorylation at the Tyr216 site.^16^ The inactivation of GSK3β might lead to a decrease in the degradation of β-catenin, thereby activating Wnt/β-catenin signaling. Previous studies demonstrated that the treatment of AR leads to a significant reduction in GSK-3β phosphorylation at Tyr216 and regulates diverse biological processes, such as cell growth,^39^ apoptosis,^40^ and survival.^41^ Besides, AR increases GSK-3β phosphorylation at Ser9 when promoting osteogenic differentiation in human adipose-derived MSCs.^31^ A similar result was obtained in the present study. Once the concentration of AR reached 5 μM, the Ser9 phosphorylation of GSK3β was significantly enhanced, but 1 μM and 2.5 μM of AR did not affect Ser9 phosphorylation of GSK3β. Meanwhile, the administration of AR resulted in an obvious decrease in the Tyr216 phosphorylation of GSK3β in a dose-dependent manner. Meanwhile, as expected, the protein level of β-catenin was elevated in rDPSCs after treated with AR. These data revealed that the activation of Wnt/β-catenin signaling might be involved in the role of AR in rDPSCs migration and osteogenic differentiation.

Moreover, our data also indicated that AR could increase the activity of the PI3K/Akt pathway in a dose-dependent manner, but displayed no obvious effect on JNK signaling in rDPSCs. Our study also revealed that the activity of the ERK1/2 in rDPSCs was obviously elevated only when the concentration of AR reached 5 μM. This suggested that a high dose of AR might further induce the activation of ERK1/2 in rDPSCs. Since our results demonstrated that AR promoted migration and osteogenic differentiation of rDPSCs once its concentration reached 2.5 μM, the subsequent experiments merely focused on the role of the PI3K/Akt pathway in the function of AR. Akt is an important member of the protein kinase family, and it is at the center of the signaling pathway, which is a biological signal transduction pathway initiated by PI3K and can be activated by many stresses. Multiple studies implicated the PI3K/Akt signaling pathway in cell proliferation and differentiation.^42^^,^^43^ Accumulating evidence has demonstrated that this pathway is involved in osteogenic differentiation.^44^^,^^45^ Besides, a recent study has revealed that the migration of DPSCs is associated with the PI3K/Akt signaling.^46^ In our study, LY294002 was used to inhibit PI3K/Akt signaling, and it was found that the treatment of LY294002 reversed the enhanced migration and osteogenic differentiation induced by AR. GSK3β is an important substrate of PI3K/Akt signaling.^47^ PI3K/Akt signaling inhibits GSK3β by enhancing its phosphorylation at the Ser9 site. Our data suggested that inhibition of GSK3β activated PI3K/Akt signaling, which was related to the role of AR in promoting migration and osteogenic differentiation of rDPSCs. Our data hinted a novel regulatory loop between GSK3β and PI3K/Akt in rDPSC migration and osteogenic differentiation.

In conclusion, the present study indicated that AR, a GSK3β inhibitor, promoted migration and osteogenic differentiation of rDPSCs *in vitro*. Activation of theβ-catenin/PI3K/Akt signaling pathway was involved in the effect of AR on migration and osteogenic differentiation of rDPSCs.

## Declaration of competing interest

The authors have no conflicts of interest relevant to this article.

## References

[1] Kucharzewski M., Rojczyk E., Wilemska-Kucharzewska K., Wilk R., Hudecki J., Los M.J. (2019). Novel trends in application of stem cells in skin wound healing. Eur J Pharmacol.

[2] Mahla R.S. (2016). Stem cells applications in regenerative medicine and disease therapeutics. Int J Cell Biol.

[3] Miana V.V., González E.A.P. (2018). Adipose tissue stem cells in regenerative medicine. Ecancermedicalscience.

[4] Grove J.E., Bruscia E., Krause D.S. (2004). Plasticity of bone marrow-derived stem cells. Stem Cell.

[5] Chen J.C., Goldhamer D.J. (2003). Skeletal muscle stem cells. Reprod Biol Endocrinol.

[6] Sunil P.M., Manikandan R., Muthumurugan, Yoithapprabhunath T.R., Sivakumar M. (2015). Harvesting dental stem cells - overview. J Pharm BioAllied Sci.

[7] Seo B.M., Miura M., Gronthos S. (2004). Investigation of multipotent postnatal stem cells from human periodontal ligament. Lancet.

[8] Yamada Y., Nakamura-Yamada S., Kusano K., Baba S. (2019). Clinical potential and current progress of dental pulp stem cells for various systemic diseases in regenerative medicine: a concise review. Int J Mol Sci.

[9] Yamada Y., Nakamura S., Ito K. (2010). A feasibility of useful cell-based therapy by bone regeneration with deciduous tooth stem cells, dental pulp stem cells, or bone-marrow-derived mesenchymal stem cells for clinical study using tissue engineering technology. Tissue Eng.

[10] Yamada Y., Ito K., Nakamura S., Ueda M., Nagasaka T. (2011). Promising cell-based therapy for bone regeneration using stem cells from deciduous teeth, dental pulp, and bone marrow. Cell Transplant.

[11] Iwata T., Yamato M., Washio K. (2018). Periodontal regeneration with autologous periodontal ligament-derived cell sheets - a safety and efficacy study in ten patients. Regen Ther.

[12] Su P., Tian Y., Yang C. (2018). Mesenchymal stem cell migration during bone formation and bone diseases therapy. Int J Mol Sci.

[13] Carbone E.J., Jiang T., Nelson C., Henry N., Lo K.W. (2014). Small molecule delivery through nanofibrous scaffolds for musculoskeletal regenerative engineering. Nanomedicine.

[14] Shi A., Heinayati A., Bao D. (2019). Small molecule inhibitor of TGF-β signaling enables robust osteogenesis of autologous GMSCs to successfully repair minipig severe maxillofacial bone defects. Stem Cell Res Ther.

[15] AlMuraikhi N., Ali D., Alshanwani A. (2018). Stem cell library screen identified ruxolitinib as regulator of osteoblastic differentiation of human skeletal stem cells. Stem Cell Res Ther.

[16] Beurel E., Grieco S.F., Jope R.S. (2015). Glycogen synthase kinase-3 (GSK3): regulation, actions, and diseases. Pharmacol Ther.

[17] Shang Y.C., Wang S.H., Xiong F. (2007). Wnt3a signaling promotes proliferation, myogenic differentiation, and migration of rat bone marrow mesenchymal stem cells. Acta Pharmacol Sin.

[18] Wang Y., Zhang X., Shao J., Liu H., Liu X., Luo E. (2017). Adiponectin regulates BMSC osteogenic differentiation and osteogenesis through the Wnt/β-catenin pathway. Sci Rep.

[19] Martinez A., Alonso M., Castro A., Pérez C., Moreno F.J. (2002). First non-ATP competitive glycogen synthase kinase 3 beta (GSK-3beta) inhibitors: thiadiazolidinones (TDZD) as potential drugs for the treatment of Alzheimer's disease. J Med Chem.

[20] Roca C., Campillo N.E. (2020). Glycogen synthase kinase 3 (GSK-3) inhibitors: a patent update (2016-2019). Expert Opin Ther Pat.

[21] Morales-García J.A., Susín C., Alonso-Gil S. (2013). Glycogen synthase kinase-3 inhibitors as potent therapeutic agents for the treatment of Parkinson disease. ACS Chem Neurosci.

[22] Leclerc S., Garnier M., Hoessel R. (2001). Indirubins inhibit glycogen synthase kinase-3 beta and CDK5/p25, two protein kinases involved in abnormal tau phosphorylation in Alzheimer's disease. A property common to most cyclin-dependent kinase inhibitors?. J Biol Chem.

[23] Bhat R., Xue Y., Berg S. (2003). Structural insights and biological effects of glycogen synthase kinase 3-specific inhibitor AR-A014418. J Biol Chem.

[24] Shakoori A., Mai W., Miyashita K. (2007). Inhibition of GSK-3 beta activity attenuates proliferation of human colon cancer cells in rodents. Cancer Sci.

[25] Kunnimalaiyaan S., Gamblin T.C., Kunnimalaiyaan M. (2015). Glycogen synthase kinase-3 inhibitor AR-A014418 suppresses pancreatic cancer cell growth via inhibition of GSK-3-mediated Notch1 expression. HPB.

[26] Alksne M., Simoliunas E., Kalvaityte M. (2019). The effect of larger than cell diameter polylactic acid surface patterns on osteogenic differentiation of rat dental pulp stem cells. J Biomed Mater Res.

[27] Vandesompele J., De Preter K., Pattyn F. (2002). Accurate normalization of real-time quantitative RT-PCR data by geometric averaging of multiple internal control genes. Genome Biol.

[28] Spyridopoulos T., Lambropoulou M., Pagonopoulou O. (2015). Regenerated nerve defects with a nerve conduit containing dental pulp stem cells in pigs: an immunohistochemical and electrophysiological evaluation. J Reconstr Microsurg.

[29] Syed-Picard F.N., Du Y., Lathrop K.L., Mann M.M., Funderburgh M.L., Funderburgh J.L. (2015). Dental pulp stem cells: a new cellular resource for corneal stromal regeneration. Stem Cells Transl Med.

[30] Gronthos S., Mankani M., Brahim J., Robey P.G., Shi S. (2000). Postnatal human dental pulp stem cells (DPSCs) in vitro and in vivo. Proc Natl Acad Sci U S A.

[31] Zhang M., Zhang P., Liu Y., Zhou Y. (2017). GSK3 inhibitor AR-A014418 promotes osteogenic differentiation of human adipose-derived stem cells via ERK and mTORC2/Akt signaling pathway. Biochem Biophys Res Commun.

[32] Shen S., Zhang Y., Zhang S. (2021). 6-bromoindirubin-3'-oxime promotes osteogenic differentiation of periodontal ligament stem cells and facilitates bone regeneration in a mouse periodontitis model. ACS Biomater Sci Eng.

[33] Huang X., Zhong L., Hendriks J., Post J.N., Karperien M. (2018). The effects of the WNT-signaling modulators BIO and PKF118-310 on the chondrogenic differentiation of human mesenchymal stem cells. Int J Mol Sci.

[34] Komori T. (2019). Regulation of proliferation, differentiation and functions of osteoblasts by Runx2. Int J Mol Sci.

[35] Takahashi T. (2011). Overexpression of Runx2 and MKP-1 stimulates transdifferentiation of 3T3-L1 preadipocytes into bone-forming osteoblasts in vitro. Calcif Tissue Int.

[36] Liu Q., Li M., Wang S., Xiao Z., Xiong Y., Wang G. (2020). Recent advances of osterix transcription factor in osteoblast differentiation and bone formation. Front Cell Dev Biol.

[37] Renn J., Winkler C. (2009). Osterix-mCherry transgenic medaka for in vivo imaging of bone formation. Dev Dynam.

[38] Majidinia M., Aghazadeh J., Jahanban-Esfahlani R., Yousefi B. (2018). The roles of Wnt/β-catenin pathway in tissue development and regenerative medicine. J Cell Physiol.

[39] Carter Y.M., Kunnimalaiyaan S., Chen H., Gamblin T.C., Kunnimalaiyaan M. (2014). Specific glycogen synthase kinase-3 inhibition reduces neuroendocrine markers and suppresses neuroblastoma cell growth. Cancer Biol Ther.

[40] Yadav A.K., Vashishta V., Joshi N., Taneja P., AR-A (2014). Used against GSK3beta downregulates expression of hnRNPA1 and SF2/ASF splicing factors. J Oncol.

[41] Darshit B.S., Ramanathan M. (2016). Activation of AKT1/GSK-3β/β-Catenin-TRIM11/Survivin pathway by novel GSK-3β inhibitor promotes neuron cell survival: study in differentiated SH-SY5Y cells in OGD model. Mol Neurobiol.

[42] Peltier J., O'Neill A., Schaffer D.V. (2007). PI3K/Akt and CREB regulate adult neural hippocampal progenitor proliferation and differentiation. Dev Neurobiol.

[43] Yu J.S., Cui W. (2016). Proliferation, survival and metabolism: the role of PI3K/AKT/mTOR signalling in pluripotency and cell fate determination. Development.

[44] Lu S.Y., Wang C.Y., Jin Y. (2017). The osteogenesis-promoting effects of alpha-lipoic acid against glucocorticoid-induced osteoporosis through the NOX4, NF-kappaB, JNK and PI3K/AKT pathways. Sci Rep.

[45] Yuan H., Zhao H., Wang J. (2019). MicroRNA let-7c-5p promotes osteogenic differentiation of dental pulp stem cells by inhibiting lipopolysaccharide-induced inflammation via HMGA2/PI3K/Akt signal blockade. Clin Exp Pharmacol Physiol.

[46] Li M., Sun X., Ma L. (2017). SDF-1/CXCR4 axis induces human dental pulp stem cell migration through FAK/PI3K/Akt and GSK3β/β-catenin pathways. Sci Rep.

[47] Yang K., Chen Z., Gao J. (2017). The key roles of GSK-3β in regulating mitochondrial activity. Cell Physiol Biochem.

