# Supplementary material for: Glycogen synthase kinase-3β inhibitor promotes the migration and osteogenic differentiation of rat dental pulp stem cells via the β-catenin/PI3K/Akt signaling pathway
Source: J Dent Sci. 2021 Oct 16;17(2):802–10. doi: 10.1016/j.jds.2021.09.035 (PMC9201544; doi:10.1016/j.jds.2021.09.035)
Supplement: Supplementary file 1 [file mmc1.docx]

**Table S1 The information of antibodies used in Western blot**

| Antibody | Manufacturers | Cat.no |
| --- | --- | --- |
| CD29 | Abcam | ab16895 |
| CD44 |  | ab189524 |
| CD90 |  | ab181469 |
| CD105 |  | ab252345 |
| CD34 |  | ab81289 |
| CD45 |  | ab10558 |
| RUNX2 |  | ab92336 |
| OPN |  | ab63856 |
| OSX |  | ab209484 |
| GSK3β |  | ab185141 |
| p-GSK3β(Tyr216) |  | ab68476 |
| p-GSK3β(Ser9) |  | ab76260 |
| β-catenin |  | ab32572 |
| PI3K |  | ab154598 |
| p-PI3K(p85α) |  | ab191606 |
| Akt |  | ab38449 |
| p-Akt (Ser473) |  | ab81283 |
| ERK1/2 |  | ab184699 |
| p-ERK1/2 |  | ab17942 |
| JNK |  | ab17557 |
| p-JNK |  | ab76572 |
| GAPDH |  | ab8245 |
| OCN | Thermo Scientific | 33-5400 |
